# Internally welded steel bars enhance the bonding between steel pipes and concrete and prevent disengagement and debonding

**DOI:** 10.1038/s41598-025-92875-z

**Published:** 2025-03-19

**Authors:** Yanfeng Deng, Haitang Zhang, Jingyao Ni, Nianchun Deng

**Affiliations:** 1 College of Civil Engineering and Architecture, Guangxi University, Nanning, 530004 China; 2 Guangxi Key Laboratory of Disaster Prevention and Engineering Safety, Guangxi University, Nanning, 530004 China

**Keywords:** Civil engineering, Structural materials

## Abstract

In this work, the bonding properties of steel pipes and concrete enhanced by internally welded steel bars are investigated, and a method to prevent debonding is proposed. A series of experiments and ultrasonic tests revealed that the internal welding of steel bars effectively improved the bond strength of the interface between the steel pipe and the concrete. A Q420qD steel pipe and C60 self-compacting compensating concrete were used in the experiment, and the filling was uniformly ensured by pumping and vacuum-assisted perfusion. The experimental results show that the concrete-filled steel tube structure has good compactness and stability, but the local voiding phenomenon indicates that the material may have voids or defects. The performance of HPB300 grade steel bars under dynamic loading is also evaluated through cube compressive strength tests and rollout tests. The tests revealed that with an increasing number of internally welded steel rings, the push-out length decreases, indicating better bonding performance. In addition, the finite element simulation and the comparison of experimental data further reveal the bond-slip characteristics of the internally welded reinforcement components of different internal forms of concrete-filled steel tubes, which provides not only important data support for the optimal design and dynamic calculation of concrete-filled steel tube structures but also a reference for subsequent material repair and reinforcement work.

## Introduction

In contemporary civil engineering, the emergence of landmark building structures challenges building materials and components. Steel tube-confined concrete (STCC), an advanced composite system, combines high-strength steel pipes and high-performance concrete, realizing complementary advantages and enhancing mechanical properties^1^. The steel pipe serves as a permanent formwork for concrete, avoiding complex construction processes. More importantly, its restraint effect on core concrete improves the compressive strength and strain of the concrete and enhances component ductility and energy dissipation. Additionally, the core concrete prevents steel pipe buckling, increases its stability, and increases the overall structure’s bearing capacity^2^.

In addition, the concrete-filled steel tube composite structure exhibited good fire resistance and corrosion resistance. The steel pipe can absorb part of the heat at high temperatures, delaying the temperature increase in the internal concrete, while the concrete can effectively protect the steel pipe from direct erosion from the external environment, and the two interact to improve the durability of the structure in harsh environments^3^. When optimizing CFST (concrete-filled steel tube) structures, ensuring the synergy between the steel tubes and the concrete and the stability of their interface is crucial for overall performance and durability. In concrete-filled steel tubular arch bridges, the mechanical properties of the CFST components affect bridge safety and service life. The bridge experiences static and dynamic forces, and interface degradation, such as debonding and emptying, can reduce the overall mechanical performance and shorten the service life^4^.

The strengthening of the bond strength of the CFST interface must be explored. The introduction test is the main means to analyze the bond-slip characteristics of the CFST interface; this test can accurately capture and quantify the changes in the bond strength of components under different loading stages and provides valuable experimental data for understanding the interface behavior On this basis, researchers have actively explored a variety of technical measures to improve the bond strength and interaction between the concrete and the steel pipe through the structural transformation of the inner wall of the steel pipe^5^. As an innovative reinforcement strategy, internally welded circular steel bars have attracted much attention because of their significant potential in improving the interfacial bond strength. Welding a circular steel bar on the inner wall increases not only the mechanical occlusion force of the contact surface but also the stress state of the concrete through the restraint effect of the steel bar to comprehensively improve the overall performance of the CFST component^6^. Although this strategy has theoretical advantages, systematic research on its application effect, optimization parameters and influence on CFST components is insufficient^7^^,^^8^.

To improve the bond strength of a CFST, researchers have recently proposed many measures, usually installing structural measures on the inner wall of the steel tube to improve the bond strength between the two materials^9,10^. Paula Fono et al.^11–13^ carried out a series of push-out tests on circular and directional CFST columns, and the results revealed that the bonding strength of the stainless steel CFST columns was greater than that of the carbon steel columns. With increasing age and cross-sectional size of the concrete, the bond strength decreases. The internal welding of steel bars is an effective measure for improving bond strength, followed by the welding of shear nails and the use of expansion pipes. Ding et al.^14^ conducted push-out tests on the roughness of the inner wall of a steel pipe, the welded longitudinal ribs and the setting of studs, and the results revealed that the rougher the inner wall is, the stronger the adhesion performance. Welding longitudinal ribs can significantly improve the bond strength, which is proportional to the rib length. The number of studs also affects the bond strength. Djerbi Tegguer et al.^15^ proposed building a pattern into a steel pipe, which can significantly improve the bond strength between the steel pipe and the concrete. Etxeberria et al.^16^ noted that the bond‒slip curve of a CFST without an additional structure is composed of an ascending section, a rapidly descending section and a residual section. Moreover, the most effective built-in measures were welded round and vertical ribs. In practical applications, many types of damage, such as debonding and hollowing, lead to decreased cohesion in concrete-filled steel tubes, and in the application of concrete-filled steel tube arch bridge engineering, the influence of vibration is a key factor^17–22^. Through many experiments and finite element simulation studies, researchers worldwide have characterized the hysteresis characteristics and ductility of concrete‒filled steel tube components and established a bending moment‒curvature hysteretic model and axial force‒displacement recovery force model of compression‒bending components, which have laid a theoretical foundation for the dynamic calculation of concrete‒filled steel tube structures^23–26^.

In view of this, this work focuses on the influence of structural measures of internally welded circular steel bars on the interface bond strength and antivibration performance of the CFST interface^27^. By constructing CFST column members with the same arch rib material as the actual engineering concrete-filled arch bridge but different internal structures and comprehensively using various research methods, such as push-out tests, comparative analyses, and numerical simulations, the influence mechanism of the internally welded steel bar on the interface bonding performance and the dynamic response of the structure can be analyzed in depth^28^. This study not only fills the current research gap but also provides a solid theoretical basis and practical reference for the design optimization, construction control, and long-term maintenance of CFST structures, which is highly important for promoting the wide application of CFST structures in bridge engineering^29^.

## Detachment and debonding test of a concrete-filled steel tubular sample

**Fig. 1:**
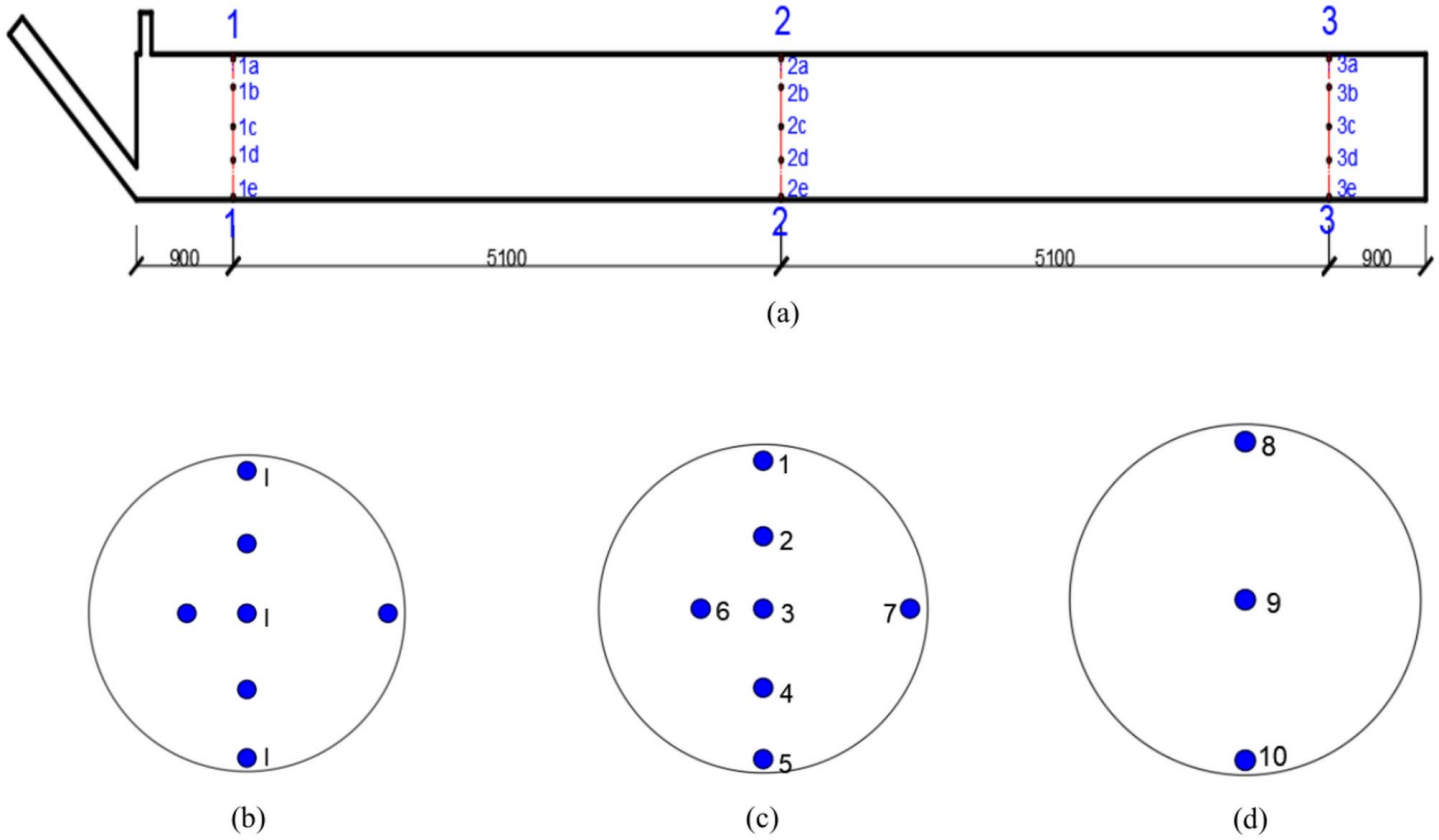
Full-scale concrete-filled steel tube sample. (**a**) Component sinusoidal strain gauge cloth setting. (**b**) The sinusoidal strain gauge is arranged in the I section. (**c**) The sinusoidal strain gauge is arranged in the II section. (**d**) Sinusoidal strain gauge arrangement III section.

For the concrete-filled steel tube sample test, a Q420qD steel pipe and C60 self-compacting compensation concrete were used. The steel pipe thickness was 32 mm, the outer diameter was 1,400 mm, and the concrete material mix ratio is shown in Table 1 The onsite full-length concrete-filled steel tube samples from the 3 tests and the full-scale concrete-filled steel tube components are shown in Fig. 1, and the strain gauge arrangement of the 3 full-scale concrete-filled steel tube samples is shown in Table 2. One Zoomlion ZLJ5180THBE truck-mounted pump was used in the test, with a theoretical maximum pressure of 28 MPa. One set of ZKB2500 vacuum pumping equipment was used, the pumping rate was 2500m^3^/h, and the slurry pipe, slurry storage cylinder and vacuum pump were connected by steel wire pipes. With pumping and vacuum-assisted perfusion, the concrete in the process of pumping jacking achieves uniform filling via the continuous flow of jacking force and the effect of removing internal bubbles.

**Table 1:** C60 self-compacting compensating concrete mix ratio (kg/m^3^).

Cement	Fly ash	Silica fume		Bulking agent
395	55	22		49
Sand	Macadam	Water	Water reducer	Microbeads
741	1067	159	11.78	27

**Table 2:** Strain gage distribution.

Location	Number	Location	Number	Location	Number
1a	2	2a	2	3a	2
1b	1	2b	1	3b	1
1c	2	2c	2	3c	2
1d	1	2d	1	3d	1

The stress distribution of each steel tube is the hoop strain of three different measuring points, and the hoop strain can reach approximately 150 με in approximately 48 h; then, there is no obvious change in nearly 120 h, and the pressure holding capacity is good. Under vacuum pumping, the concrete has a high degree of compactness, the internal expansion benefit is transformed into effective prestress expansion, and the measured 168 h prestress effect is well maintained, all of which are approximately 1.5 MPa, meeting the demand for micropressure maintenance.

**Fig. 2:**
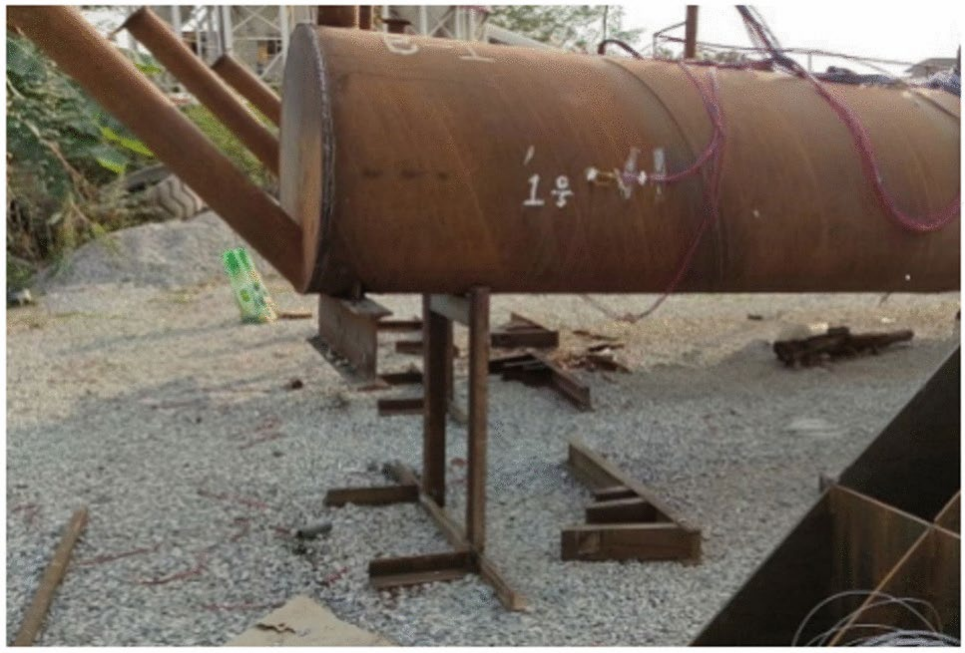
Sample of full-scale concrete-filled steel tubular section.

**Fig. 3:**
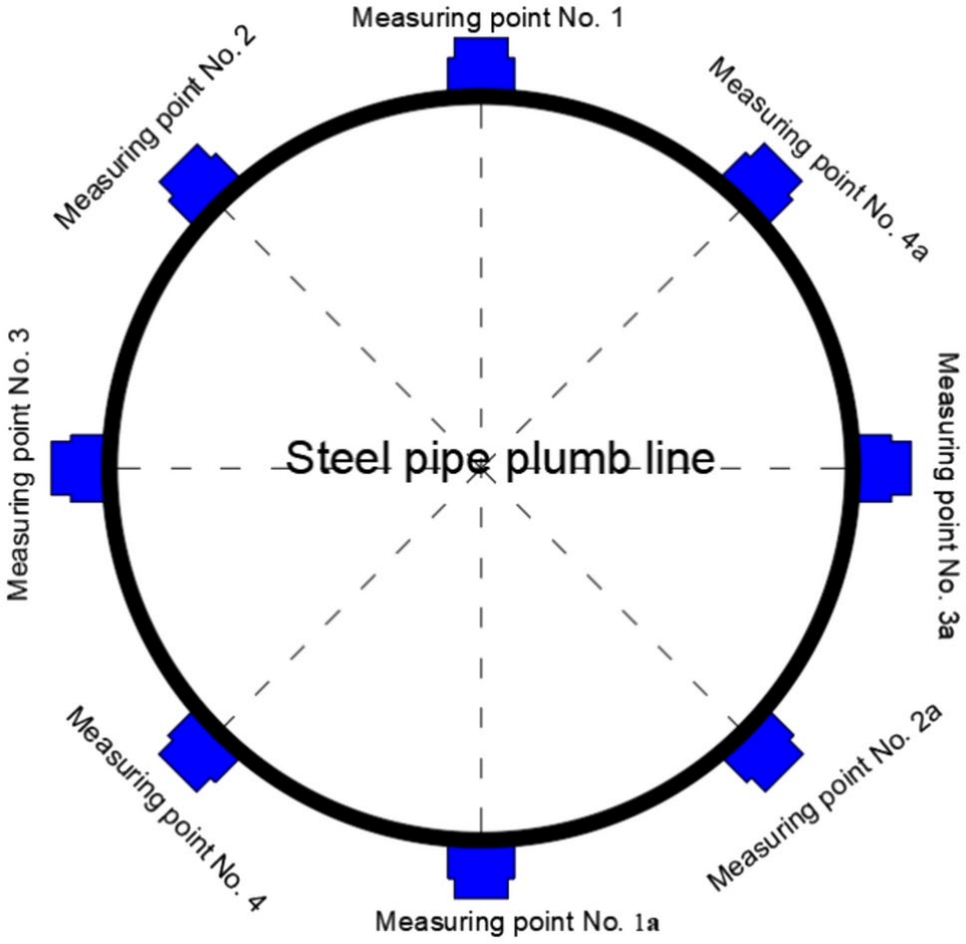
Ultrasonic measurement cross-sectional layout.

**Fig. 4:**
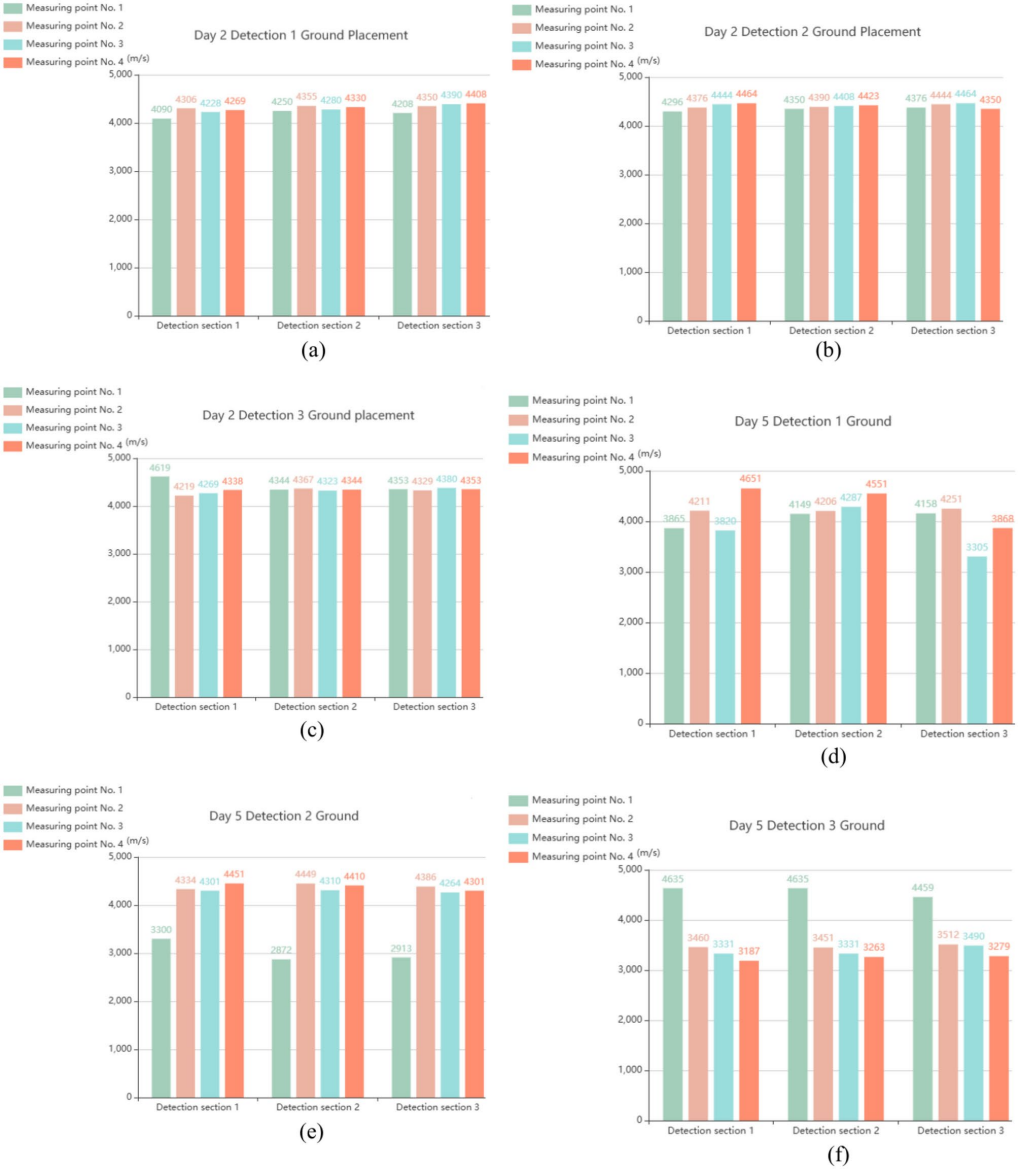

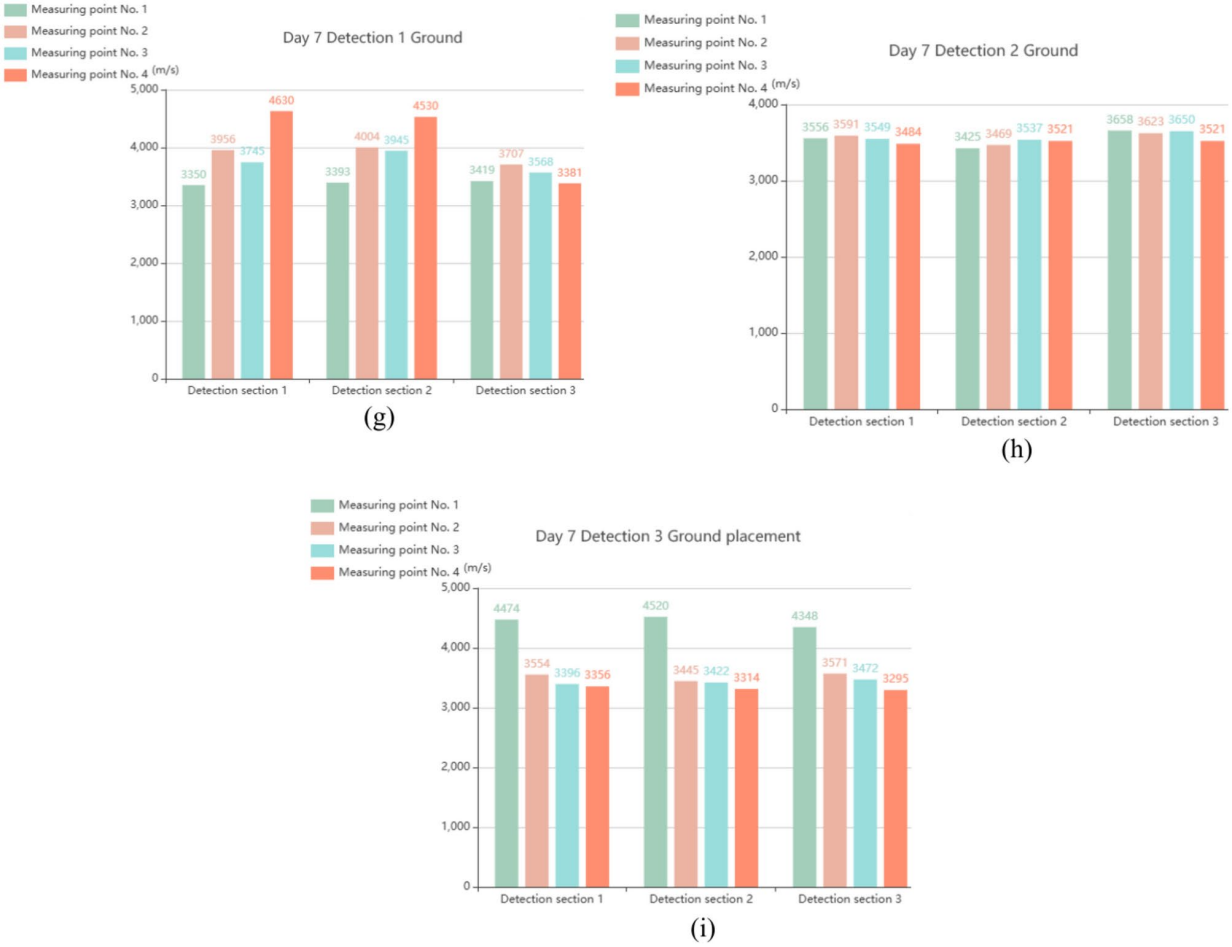
Ultrasonic test results at the test point. (**a**) Day 2: Detection 1 ground placement, (**b**) Day 2: Detection 2 ground placement, (**c**) Day 2: Detection 3 ground placement, (**d**) Day 5: Detection 1 ground placement, (**e**) Day 5: Detection 2 ground placement, (**f**) Day 5: Detection 3 ground placement, (**g**) Day 7: Detection 1 ground placement, (**h**) Day 7: Detection 2 ground placement. (**i**) Day 7: Detection 3 ground placement.

Figure 2 shows the sample of full-scale concrete-filled steel tubular section. In the process of onsite testing of concrete-filled steel tubes, the debonding of the core concrete in the concrete-filled steel tube structure is nondestructively tested via the percussion method, the quality assessment standard of the concrete-filled steel tube at a single point is ultrasonic testing, the wave velocity is determined, and the concrete-filled steel tube has good compactness. Using a Zhibolian ZBL-U510 nonmetallic ultrasonic detector, the ultrasonic measurement cross-sectional layout is shown in Fig. 3. The ultrasonic detection results of the measurement points on the 2nd, 5th and 7th days are shown in Fig. 4, and the results are well evaluated.

**Fig. 5:**
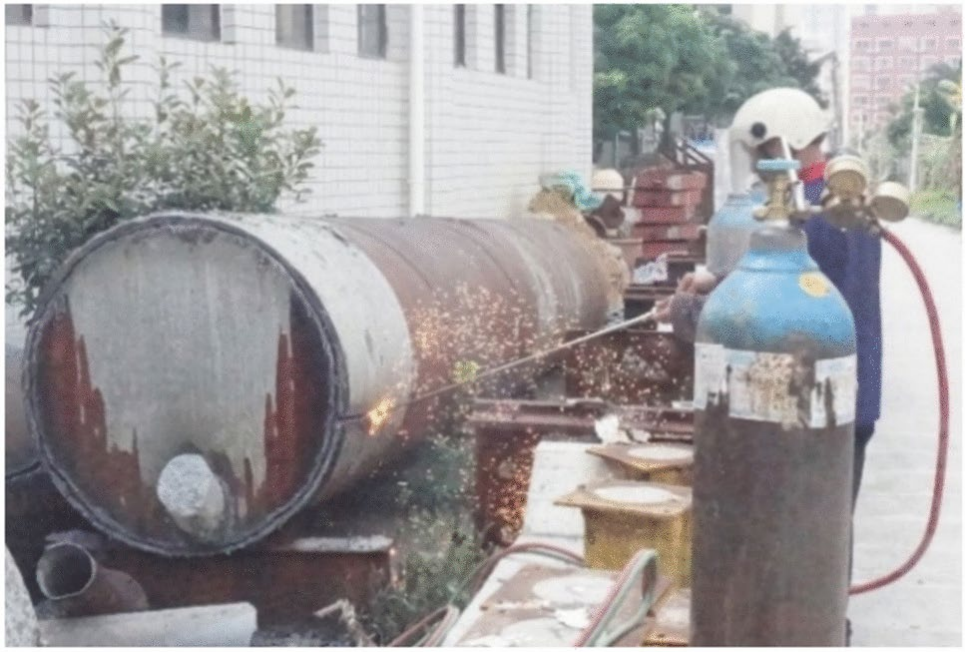
Full-scale concrete-filled steel tube section: flame to cut the cutting tool.

**Fig. 6:**
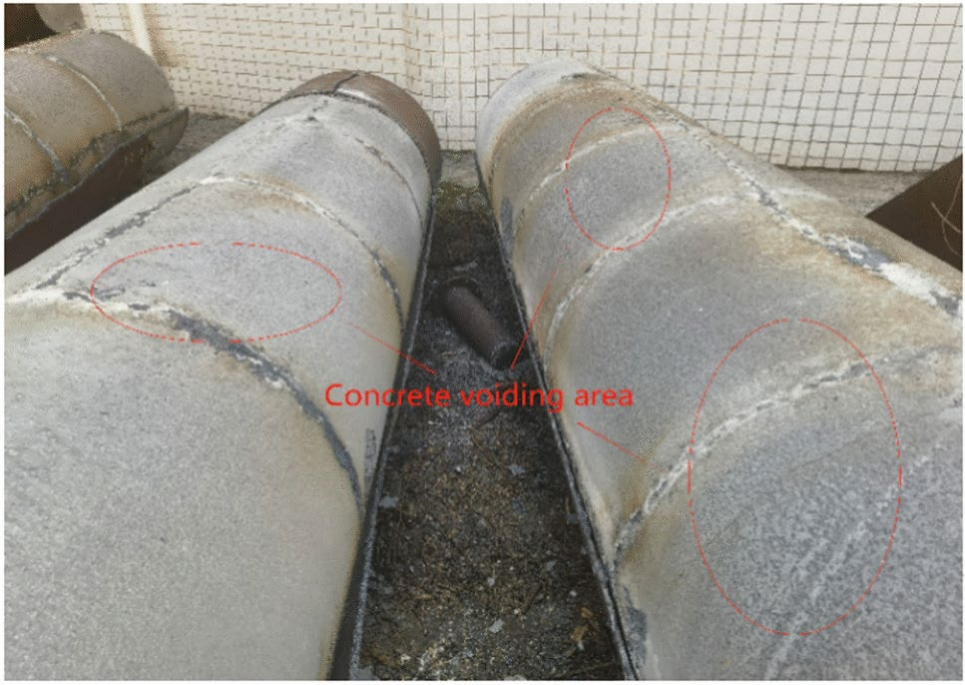
Hollowing area of the full-scale concrete-filled steel tube section.

**Fig. 7:**
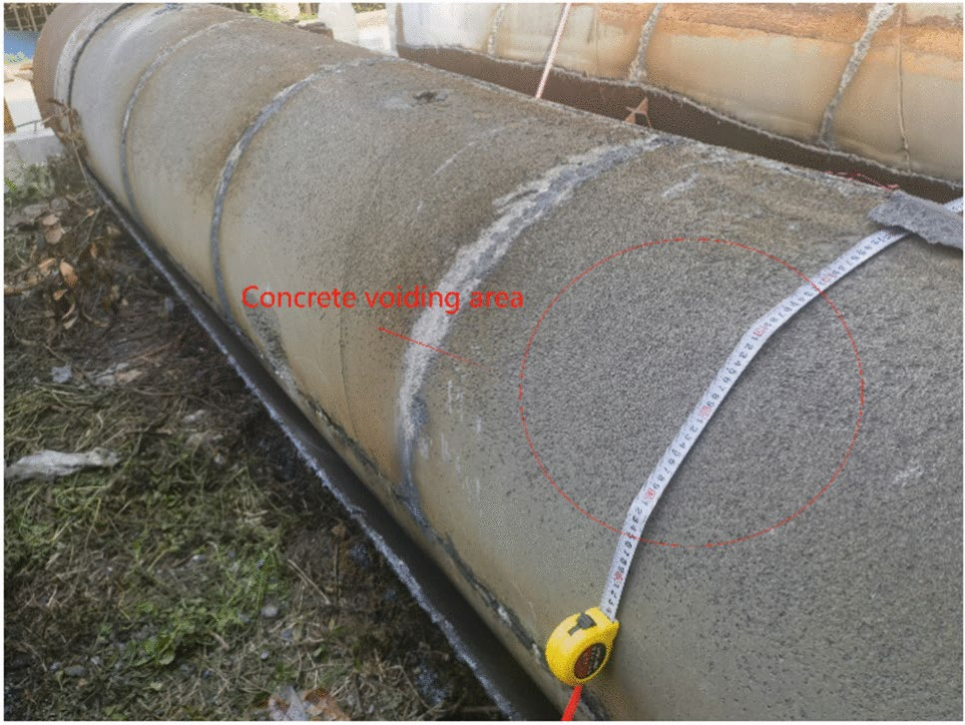
Full- scale steel pipe concrete dehydration.

**Fig. 8:**
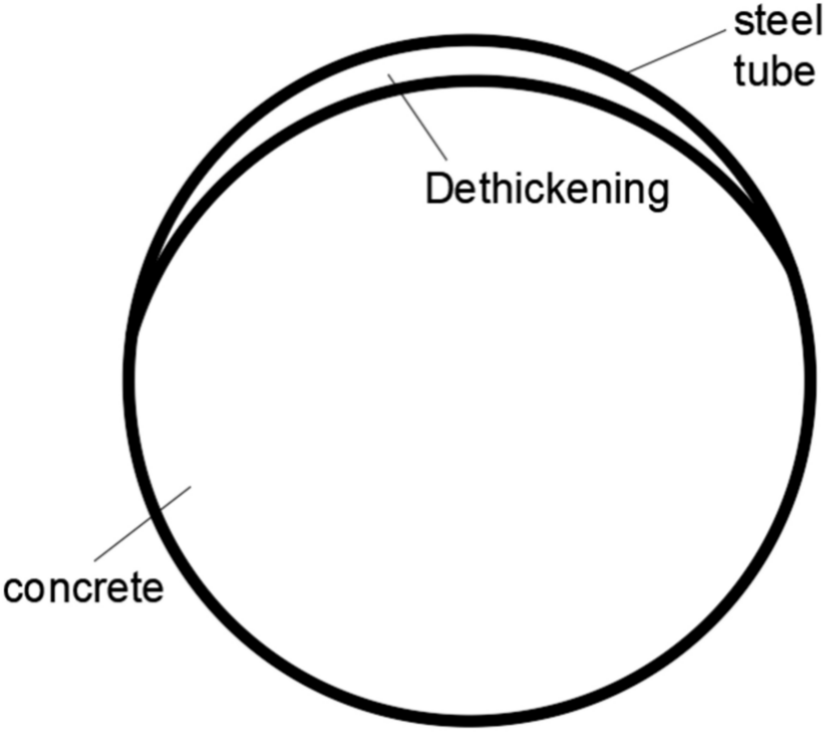
Debonding model of concrete-filled steel tube.

**Fig. 9:**
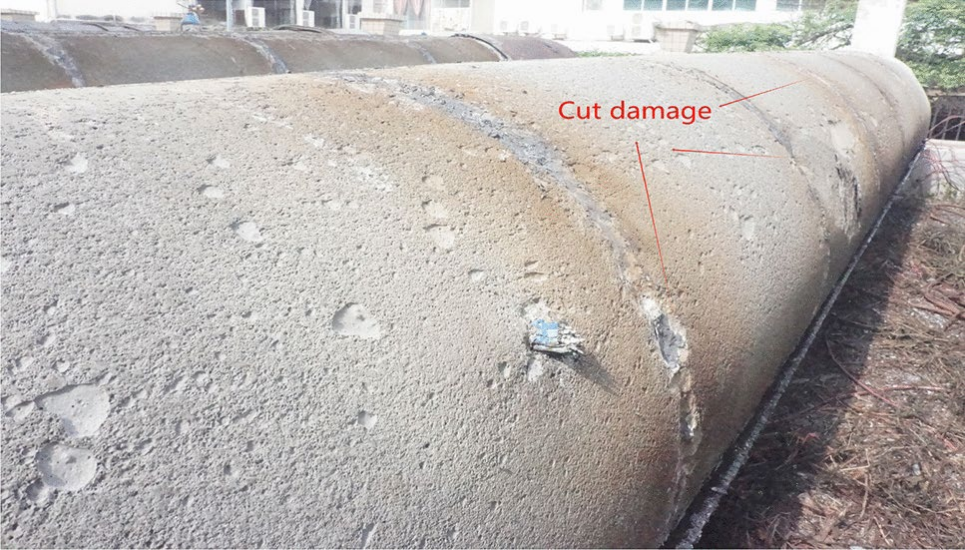
Cutting injury.

After the full-scale CFST test, the flame is cut, indicating that approximately 1/3 of the upper part of the concrete-filled steel tube interface is empty, as shown in Figs. 5, 6, 7 and 8. The debonding of a concrete-filled steel tube is a phenomenon in which two materials with different shrinkage properties have different shrinkage and bubble conditions under the action of the external environment and time, which leads to detachment of the contact interface. The core concrete is radially separated from the pipe wall, and the debonding model of the concrete-filled steel tube is shown in Fig. 8. The above voiding is due to bubbles, and hollowing affects the common force; the concrete is damaged during flame cutting to study the emptying degree of the poured concrete, as shown in Fig. 9.

Figure 4 presents the ultrasonic test results of the CFST samples on Days 2, 5, and 7, highlighting the progressive improvement in the steel–concrete interface bonding quality. On Day 2, the ultrasonic wave velocity varied, indicating incomplete compaction of the interface. By Day 5, the bonding improved as the concrete hardened, with more uniform wave velocities observed. By Day 7, the interface reached a stable and dense state, demonstrating a significant increase in the strength and bonding quality of the concrete. However, the tests revealed voids in the upper one-third of the steel tube, likely caused by trapped air during casting, resulting in localized bonding deficiencies. Overall, the results confirm the effectiveness of vacuum-assisted casting in improving concrete compactness and provide insights for optimizing CFST structural performance.

## Push-out test of internally welded reinforced steel concrete-filled steel tubes

### Experimental schemes

The experimental design involves four different forms of concrete-filled steel tubes with internally welded steel bars. For all the steel pipes, the diameter is 370 mm, the thickness is 10 mm, the height is 550 mm, and the material is Q235 steel. The core concrete is C60 self-compacting concrete, which is consistent with the actual engineering situation. The diameter of the inner welded steel bar is 10 mm, the material is HPB300, and this type of steel bar is a connector suitable for bearing the dynamic load of the concrete-filled steel tube composite structure. During pouring, a distance of 50 mm is reserved at the bottom of the concrete, and the interfacial bonding length of the component is 500 mm. The remaining sample parameters are shown in Table 3.

**Table 3:** Sample parameters.

Sample number	Internal reinforcement form	Stirrup spacing (mm)
R0	–	–
R1	One round tendon	250
R2	Two round tendons	160
R3	Three round tendons	125

**Fig. 10:**
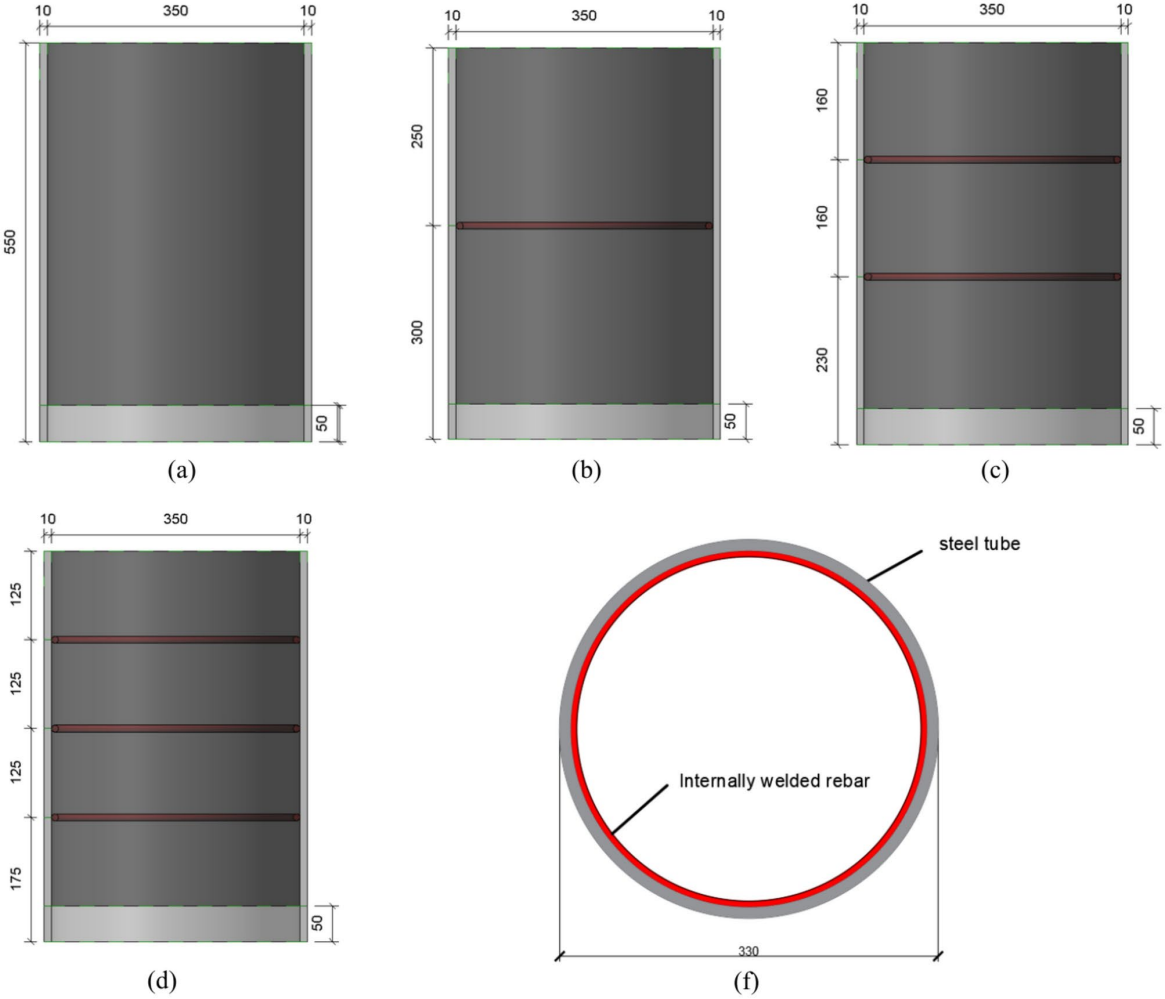
Design diagram of the sample construction measures (mm). (**a**) R0 cross-section, (**b**) R1 cross-section, (**c**) R2 cross-section, (**d**) R3 cross-section, (**f**) Top view.

**Fig. 11:**
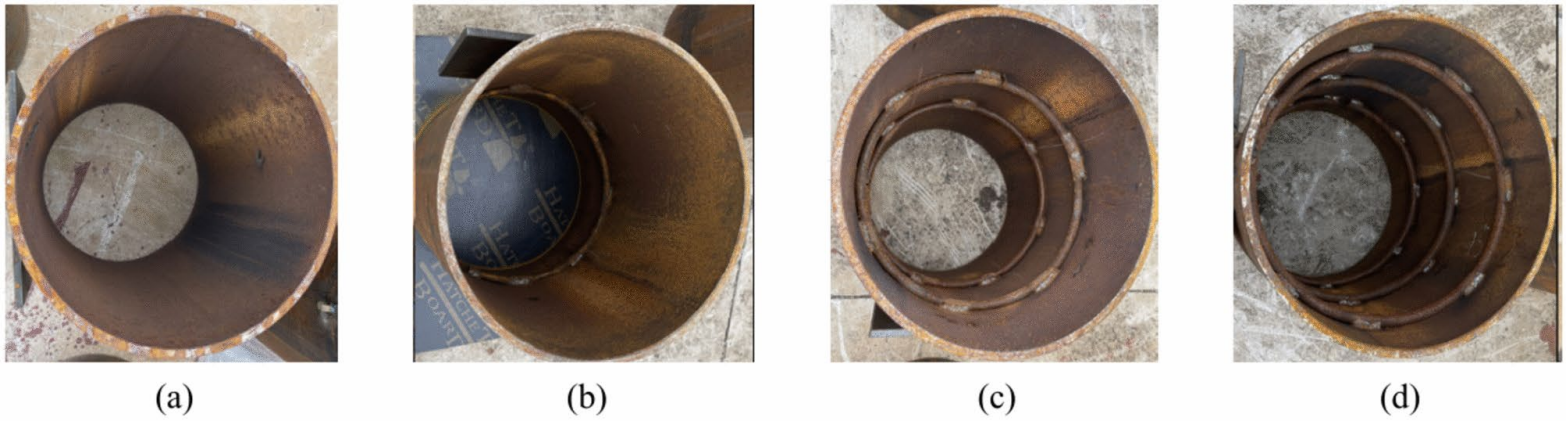
Actual photo of the steel pipe sample. (**a**) R0, (**b**) R1, (**c**) R2, (**d**) R3.

In Table 3, R0 is the component without the form of internal reinforcement; R1, R2 and R3 are provided with one, two and three circular reinforcements, respectively. Figures 10 and 11 are the design sections of the component and the actual photo, respectively. HPB300 grade steel bars are used for the steel bars welded in the steel pipes, and Table 4 shows the mechanical properties of the steel bar materials.

**Table 4:** Mechanical properties of the steel materials.

Location	Size (mm)	Fy (MPa)	Fu (MPa)	Es (MPa)	δ (%)
Steel tube	10	235	380	2.00*10E5	25
Internally welded rebar	10	300	425	1.98*10E5	10

To assess the material properties of the C60 self-compacting concrete used in the test, the cubic compressive strength and axial compressive strength of the CFST are tested according to the "Standard for Test Methods for Mechanical Properties of Ordinary Concrete" (GB/T 50081-2002), while the concrete-filled steel tube components are poured, resulting in a total of 9 test blocks. After 28 days of curing, the compressive strength was tested on a 200 t hydraulic pressure testing machine in the building materials laboratory of Guangxi University, and the load was adjusted manually.

**Fig. 12:**
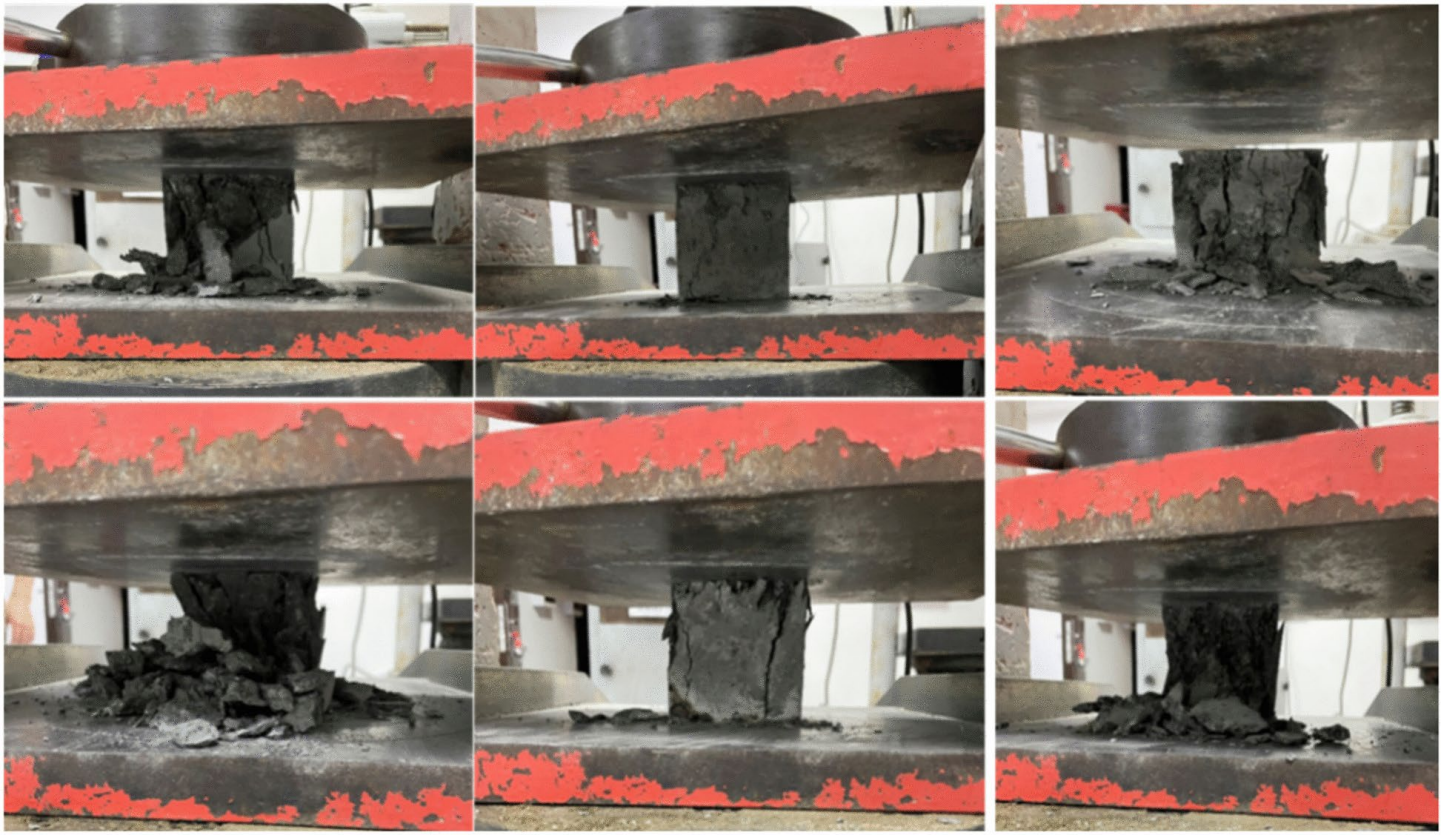
Cubic compressive strength test.

In accordance with the requirements of the "Standard for Test Methods for Physical and Mechanical Properties of Concrete" (GB/T 50081-2019), the compressive strength of a concrete test cube with a strength grade greater than C60 should be a standard cube sample with a side length of 150 mm. If a nonstandard cube sample with a side length of 100 mm is used, the size conversion coefficient should be determined by testing. The compressive strength of the cube is tested with a 100 mm*100 mm*100 mm test block, and the conversion coefficient of the conversion standard test block should be multiplied by 0.95. A schematic of the test is shown in Fig. 12, and the test results are shown in Table 5.

**Table 5:** Cube test results.

Sample type	Group I	Group II	Group III	Group IV	Group V	Group VI	Average value
Cube compressive strength (MPa)	67.7	68.4	72.6	69.8	73.4	72.8	70.7
Conversion to the standard value (MPa)	64.3	64.9	68.9	66.3	69.7	69.1	67.2

In the cubic compressive—strength test, there is no obvious change in the appearance of the specimen at the initial stage. As the compression—testing machine continuously and uniformly applies a vertical load, fine cracks begin to appear on the surface of the specimen. These cracks first initiate at the corners or stress—concentration areas of the specimen. Subsequently, the cracks extend and propagate into the interior of the specimen at a certain angle, and a small amount of fine material debris falls off.

As the load further increases, the cracks rapidly multiply and connect with each other to form an obvious crack network. At this time, the amount of material debris spalling from the surface of the specimen gradually increases, and the integrity of the specimen is greatly damaged.

When the load approaches the ultimate compressive strength of the material, the cracks on the surface of the specimen expand sharply, a large number of material particles fall off from the specimen, and the specimen makes an obvious breaking sound. Finally, the specimen is crushed into several pieces, and the core material is also crushed. The specimen completely loses its bearing capacity, presenting typical compression—failure characteristics. The whole process intuitively shows the whole process of the material from elastic deformation to plastic deformation and then to failure under the action of pressure.

**Fig. 13:**
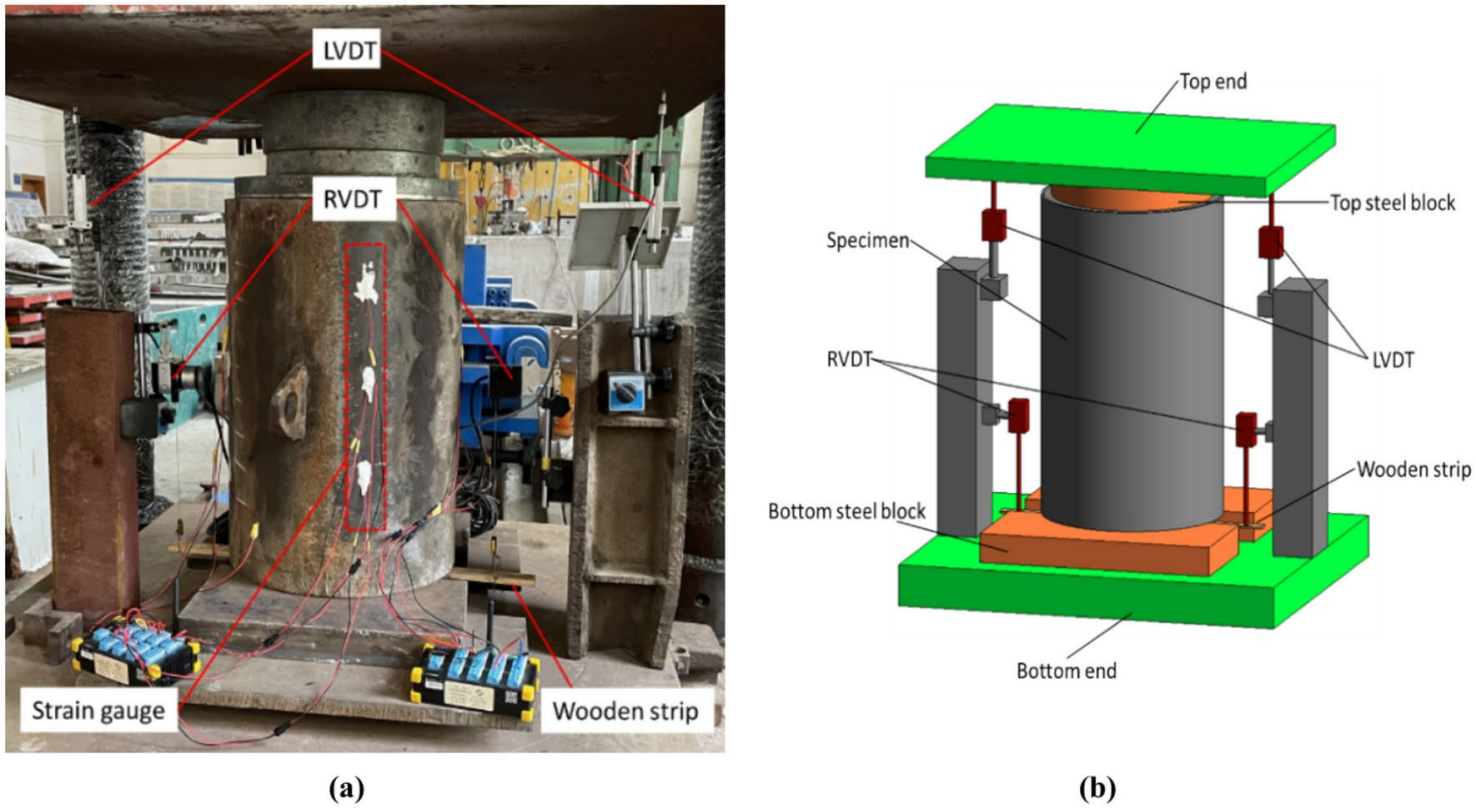
Test layout. (**a**) Test site layout, (**b**) Three-dimensional schematic of the test.

The concrete-filled steel tube launch test was carried out on the 1,000-ton press test bench of the School of Civil Engineering and Architecture of Guangxi University. The reserved end of the test member is placed on the cushion block at the bottom facing downward, the wooden block and the wooden strip are fixed at the bottom of the core concrete of the reserved end, the upper end of the wooden block is tightly bonded with the core concrete, and the lower end is fixed along the wooden strip. The joint displacement of the wood strips and the bottom of the core concrete is guaranteed, which is used to measure the slip value of the concrete. A circular steel block slightly smaller than the diameter of the core concrete is placed at the upper end of the sample, and a load is applied from above. The upper end of the test block is the loading end, and the lower end of the test block is the free end. A schematic of the test is shown in Fig. 13.

Before the start of the test, the loading end is controlled to descend and contact the sample, and the output value of the test console is an indicator. At this time, the test loading end and the free end are in full contact with the sample to eliminate the influence of virtual displacement. The position of each displacement gauge is adjusted, the plane is adjusted, the strain gauge and displacement gauge indications are clear, and the test starts. The loading process is controlled by the displacement, the loading rate is maintained at 0.5 mm/min, and the loading process stops when the loading displacement value reaches 40 mm.

### Measured point arrangements

The main parameters of the test are the axial strain value of the outer wall of the steel pipe, the slip value of the core concrete, and the displacement value of the loading end and push-out end of the sample, in which the loading displacement of the press and the push-out force of the sample are read and exported by the control software.

**Fig. 14:**
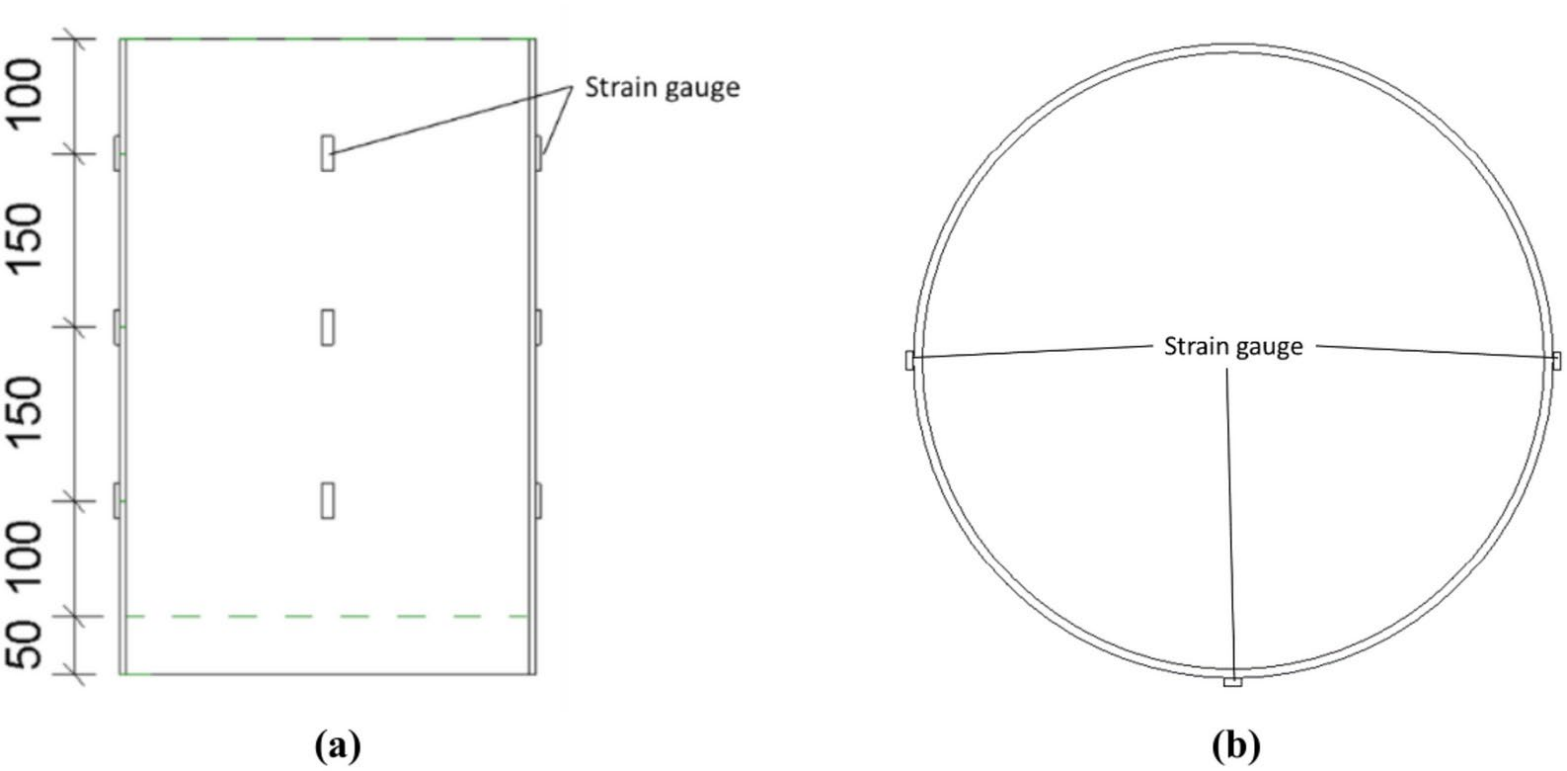
Layout of measurement points. (**a**) Front view, (**b**) Top view.

A total of four displacement sensors were set up in the test, including two pointer displacement sensors (LVDTs), which were arranged at the loading end to measure the displacement value of the loading end. Two wire pull displacement meters (RVDTs) convert changes in angular displacement to linear displacement by means of winding wheels, wire pulls, and wire-actuated encoders; it is connected to a wooden strip that is synchronized with the core concrete, and the real-time slip value of the core concrete is read by measuring the downward displacement value of the wooden strip. The tension line is perpendicular to the wooden strip when it is arranged to reduce error. The displacement gauge layout diagram is shown in Fig. 14.

The strain measurement points are arranged on the outer wall of the steel pipe, and 3 columns are set up, with 3 measurement points in each column, for a total of 9 measurement points. Each measurement point was polished with a grinding wheel to remove rust and then polished with sandpaper and wiped with alcohol. A strain gauge with a length of approximately 3 cm is spot-welded to the test point, and the sample is tightly fitted and protected with silicone. For the strain gauge measurements, a DH3819 wireless static strain test system is used. The layout of the measuring points is shown in Fig. 14.

### Damage modes

During the test, with the exception of one steel pipe that failed, the test phenomena of the remaining three steel pipes followed the same rules. At the initial stage of loading, the sample emits a “zizi” sound; at this time, the upper core concrete is compressed, the steel pipe wall is rubbed, and the bond-slip force is primarily based on the mechanical connection force, supplemented by the cementing force and the occlusion force. With increasing loading displacement, the pressure of the component gradually increases, and the fracture of the welding point of the inner welded steel bar and the steel pipe wall can be heard to make a “bang” sound. At this time, the adhesion force depends on the mechanical occlusion force of the undamaged inner welded steel bar and the concrete and the friction force of the core concrete and the steel pipe wall. Because the weld joint of the inner welded steel bar is gradually broken, the mechanical occlusion force generated is also gradually withdrawn from the work, and the concrete slip value increases linearly with increasing load. When the “bang” sound disappears, a “zizi” sound can be heard loudly, which indicates that the welding joints of the inner welded steel bar are dewelded and withdrawn from the work, and the adhesion force depends on the friction between the core concrete and the inner welded steel bar and the steel pipe wall. The slip value increases rapidly until the loading process ends^30^.

**Fig. 15:**
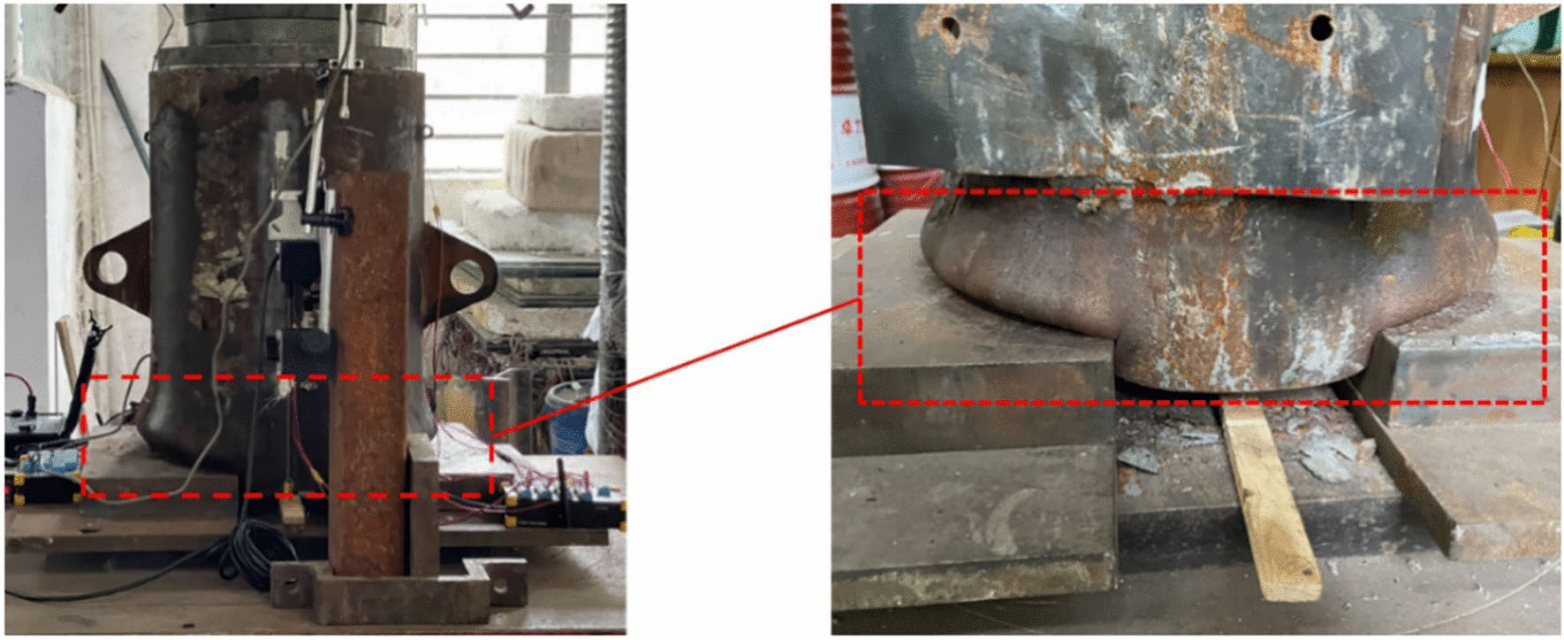
Steel pipe failure diagram.

During the test, the lower end of the external steel pipe of the S-R3 component was damaged, and the pressure of the whole component exceeded 3,200 kN after the displacement value of the loading end reached 10 mm. In the process of testing, a “bang” sound is rare because the contact area between the inner welded steel bar and the inner wall of the steel pipe in the component increases, the welding joints increase, the joint mechanical occlusion force increases, and the welding joints rarely break. However, owing to the reserved 50 mm unpoured concrete at the lower end of the steel pipe, the steel pipe wall alone cannot withstand the pressure and is damaged. The failure diagram of the steel pipe is shown in Fig. 15.

**Fig. 16:**
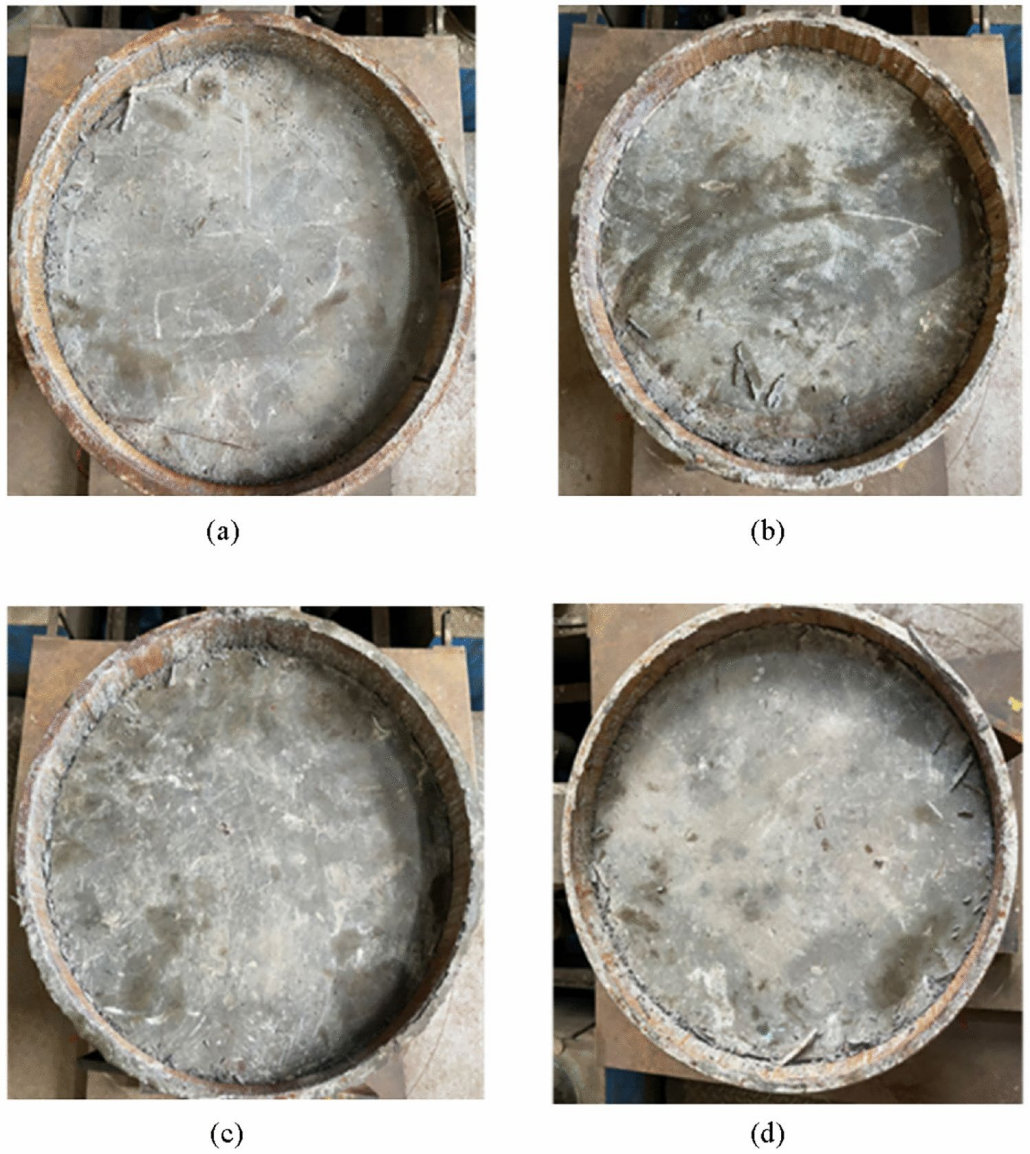
Destruction diagram of the loading end. (**a**) R0, (**b**) R1, (**c**) R2, (**d**) R3.

After the test, the core concrete is pushed out, resulting in more scratches on the inner wall of the steel pipe. The center of the core concrete is relatively flat, but there are concrete debris around the steel pipe wall, and the number of fragments in the sample with internally welded steel structures is significantly greater than that in the sample without steel bars. As shown in Fig. 16.

The ejection length at the final loading end is measured, and the ejection length is shown in Table 6 With increasing number of rings of internally welded reinforcement, the push-out length gradually decreases.

**Table 6:** Rollout length.

Sample number	R0	R1	R2	R3
Rollout length (mm)	35.4	34.8	33.6	32.4

**Fig. 17:**
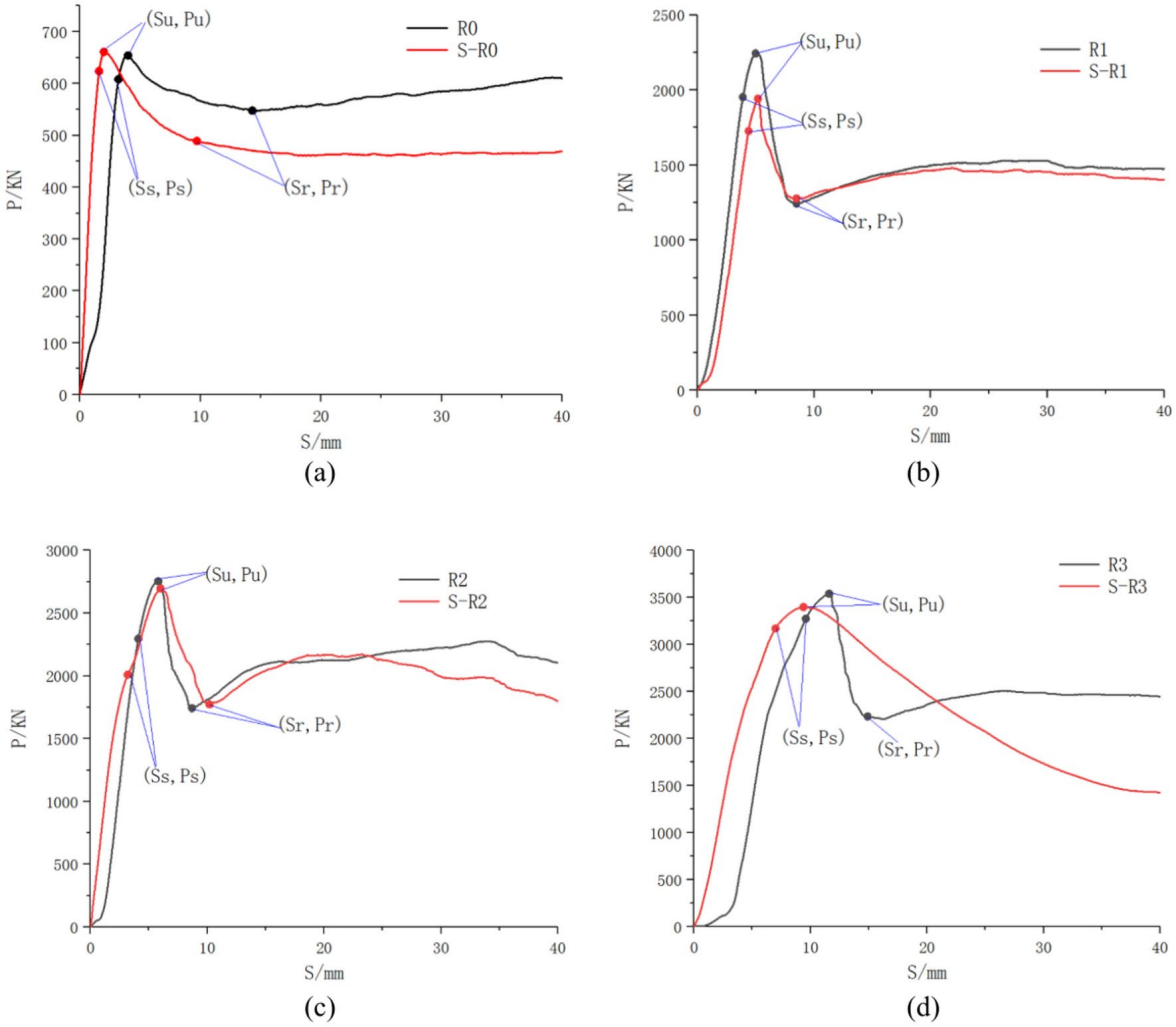
Load‒displacement (P‒S) curve. (**a**) R0, (**b**) R1, (**c**) R2, (**d**) R3.

The load displacement curves for each component are drawn separately, as shown in Fig. 17. The P‒S curves of each component are divided into three stages: ascending, descending, and residual. The descending section represents a decrease in the bond-slip performance of the component.

To describe the P‒S curve in more detail, three feature points were identified on each curve: the initial point (Ss, Ps) is the starting point where the bond slip begins to develop rapidly, the peak point (Su, Pu) is the maximum load position point in the slip process, the residual point (Sr, Pr) is the end point of the descending section after the peak, α1 is the ratio of Ps to Pu, and α2 is the ratio of Pr to Pu. Among the three failure members, the yield failure of the steel pipe has occurred after the peak point has reached, so the descending section and the residual section do not follow the rules of the bond‒slip curve, and the residual point is not set here for comparison. The eigenvalues in each P‒S curve are listed in Table 7.

**Table 7:** Eigenvalues of the load‒slip curves.

Samples	${P}_{s}$/kN	${P}_{u}$/kN	${P}_{r}$/kN	${S}_{s}$/mm	${S}_{u}$/mm	${S}_{r}$/mm	${\alpha }_{1}$	${\alpha }_{2}$
R0	608.1	654.2	547.5	3.2	4	14.4	0.93	0.84
R1	1952.5	2254.5	1244.5	3.9	5.1	8.5	0.87	0.55
R2	2295	2762.5	1746.5	4.1	5.7	8.8	0.83	0.63
R3	3274.5	3541	2235	9.6	11.5	14.9	0.92	0.63

The load curves in Fig. 17 show the load‒slip relationships for different samples. As the loading displacement increases, the curves undergo three stages, namely, ascending, descending, and residual, indicating that the bonding interface demonstrates strong adhesive forces in the initial phase, whereas friction dominates in the later stages to maintain bonding performance.

The data in Table 7 show that increasing the number of welded steel reinforcement rings significantly enhances the load-bearing capacity and slip resistance of the bonding interface. The peak slip value of sample R3 reaches 11.5 mm, and its residual slip value (14.9 mm) indicates strong anti-slip performance under large displacements. Additionally, the residual load ratio of the R3 sample (a_2_ = 0.63) exceeds that of the R1 sample, demonstrating more stable frictional forces at the interface.

Internally welded steel reinforcements significantly improve the bonding performance of CFST structures, with the R3 sample achieving the best results. This finding indicates that welding three reinforcement rings optimizes the bonding strength and anti-slip capacity of the interface. These findings provide valuable insights for enhancing CFST structures under dynamic loads and long-term service conditions, particularly in seismic and bridge engineering applications.

**Fig. 18:**
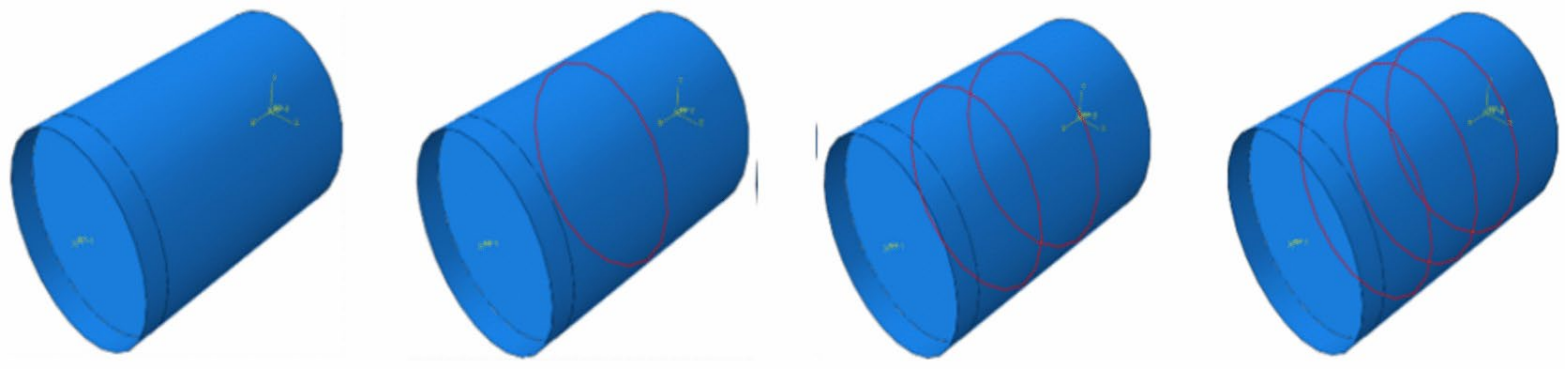
Finite element model (R0, R1, R2, and R3 from left to right).

**Fig. 19:**
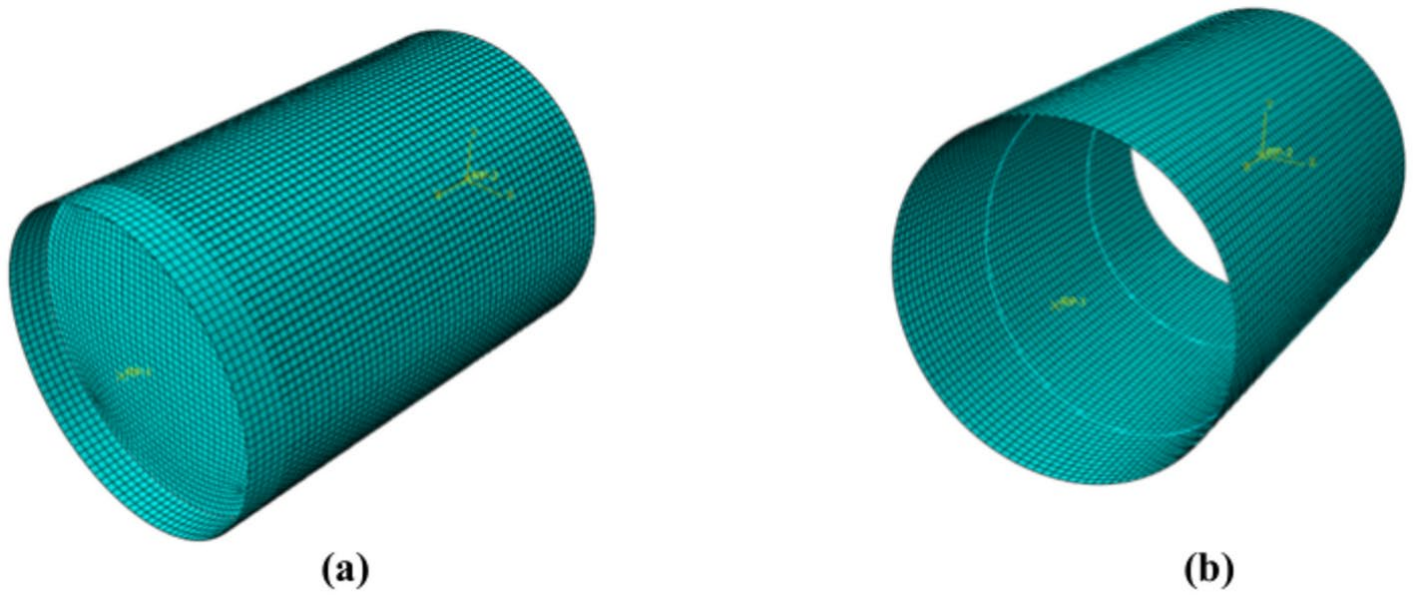
Model meshing. (**a**) Whole sample, (**b**) Steel pipe walls.

ABAQUS software is used to establish a group of four components with different forms of internally welded steel bars, and the specific size parameters of each component in the simulation process are consistent with the actual situation. In the finite element model, the steel tube and concrete interface use bonded contacts to simulate their interoperability. The steel pipe is established by the four-node shell element S4R, the core concrete is established by the eight-node three-dimensional solid element C3D8, the steel bar is established by the two-node, linear, three-dimensional truss element T3D2, and the end plates at both ends are coupled to the reference points at both ends by setting rigid body constraints in the interaction module to make them rigid bodies, as shown in Fig. 18. The meshing of the model is shown in Fig. 19. The boundary conditions and loading scheme are set in ABAQUS software, and the edge of the steel pipe is completely fixed at one end of the steel pipe with a gap in the model according to the test situation in this paper. Its displacement is limited in the x, y, and z directions, the concrete surface at the other end serves as the loading end, and a displacement load of 40 mm is applied to the concrete surface of the loading end along the positive direction of the z-axis in the simulation process^31^.

Based on these model establishment and loading methods, four different internal forms of concrete-filled steel-filled steel tubes are analyzed and calculated.

**Fig. 20:**
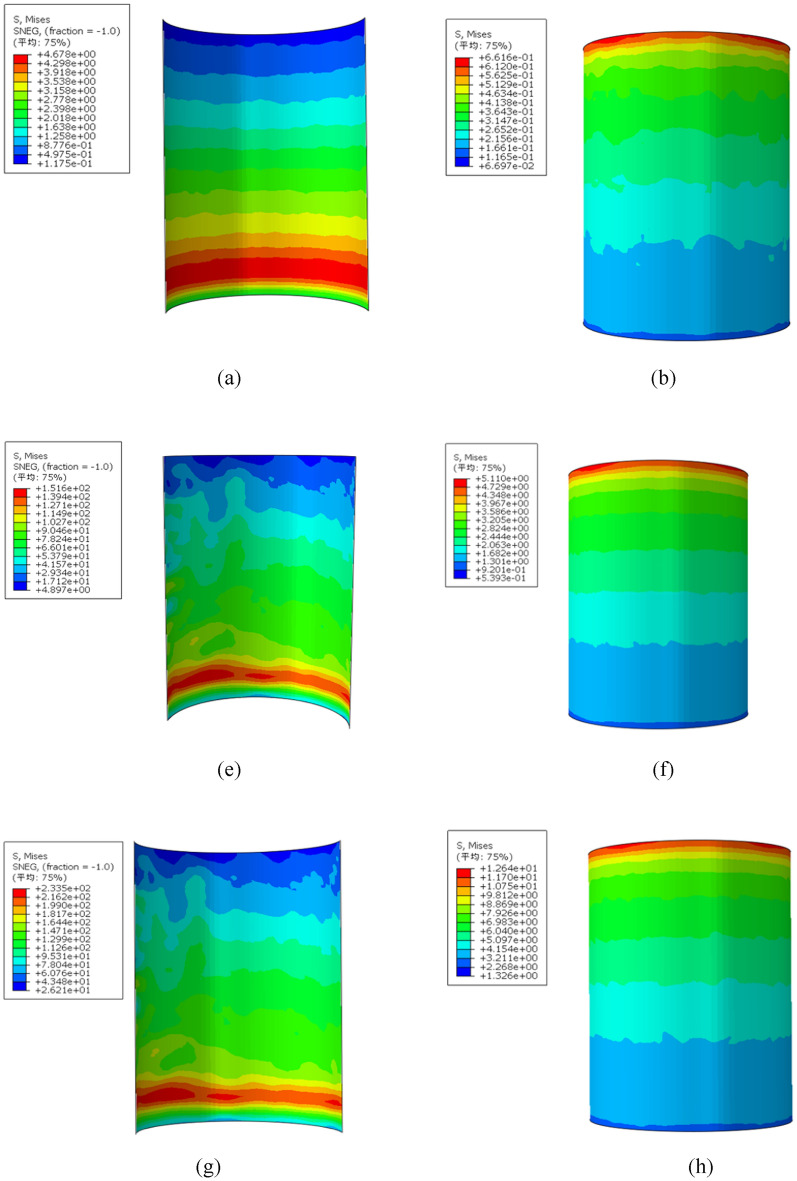
Equivalent stress contour of each component. (**a**) Stress contour diagram of the inner wall profile of the R0 steel pipe, (**b**) Stress contour diagram of the R0 concrete. (**e**) Stress contour diagram of the inner wall profile of the R2 steel pipe. (**f**) Stress contour diagram of the R2 concrete. (**g**) Stress contour of the inner wall profile of the R3 steel pipe. (**h**) Stress contour of the R3 concrete.

According to the displacement curve of the test load, when the loading displacement of each component is 15 mm, the interfacial cohesion of each component has passed the peak point and is in the stable stage, so the analysis is selected when the displacement is loaded at 15 mm. The equivalent stress contour diagram of the inner wall of the steel pipe and the surface of the core concrete of each component is shown in Fig. 20, where the loading displacement value of each component is 15 mm. The maximum and minimum values of the stress of the inner wall of the steel pipe and the surface of the core concrete of each model are obtained, as shown in Table 8.

**Table 8:** Stress values of the inner wall of the steel pipe and the concrete (MPa).

Sample number	Maximum stress of the steel pipe	Minimum stress of the steel pipe	Maximum stress of the concrete	Minimum stress of the concrete
R0	4.678	0.1175	0.6616	0.06697
R1	151.6	4.897	5.11	0.5393
R2	217.1	15.76	11.17	1.182
R3	233.5	26.21	12.64	1.326

When the loading displacement reaches 15 mm, the maximum stress value of the inner wall of the steel pipe of the R1 member with one ring of steel bars is 32.4 times greater than that of the R0 member without the structural measures of the internally welded steel bar, and the maximum stress value of the core concrete surface is increased by 7.72 times. With increasing number of welded steel rings, the bonding stress of the component increases, and the structural measures of welding the three-ring steel bar can significantly increase the bonding stress of the component^32^.

## Optimization of concrete-filled steel tubes reinforced with internally welded steel bars

**Fig. 21:**
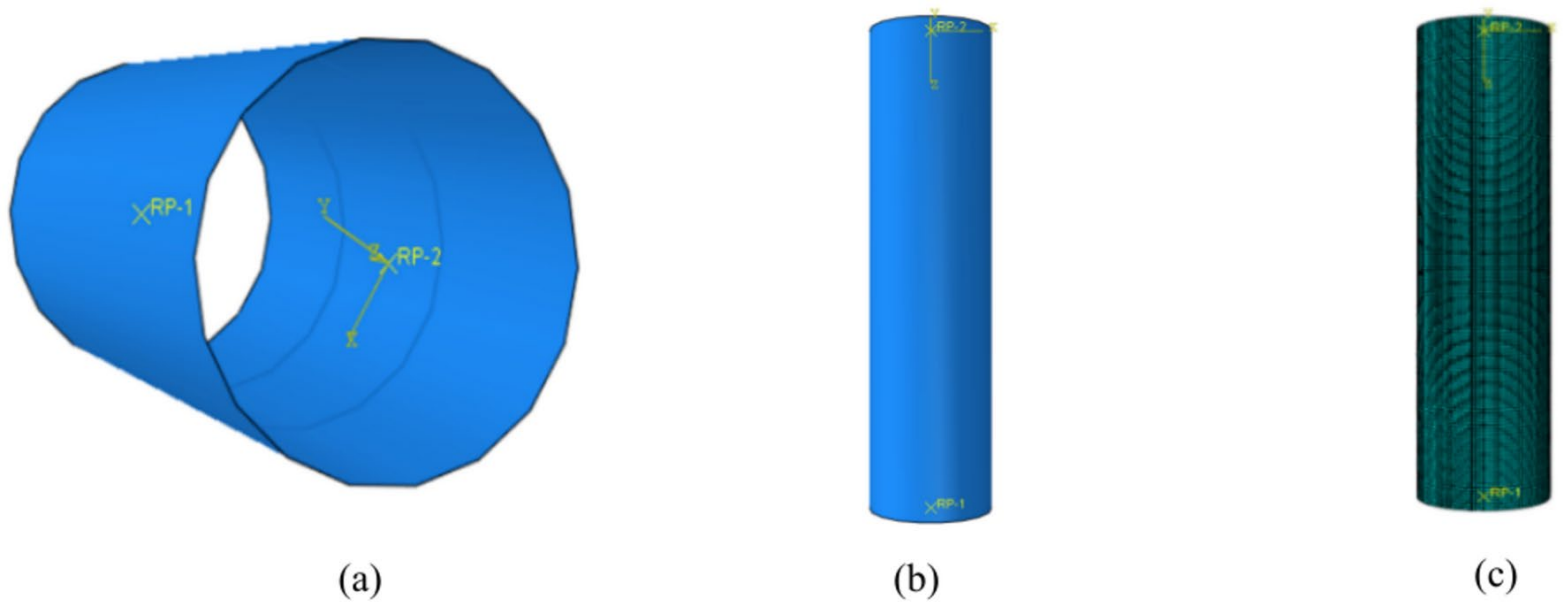
Example of sample finite element model. (**a**) Steel tube, (**b**) Whole component, (**c**) Meshing.

Owing to the increase in the number of internally welded steel bars and spacing design, the height of the concrete-filled steel tube components is increased, the height of the steel tube is increased from the original 550 mm to 1,000 mm for calculation, and the diameter of each welded steel bar is also 10 mm. Moreover, to save computing power, the calculation is stopped when the calculation displacement is loaded to 20 mm. The finite element model and meshing examples of all the components are shown in Fig. 21, and the design of each optimization comparative analysis sample is as shown.

The structures of 0, 1, 2, 3, 4, 5 and 8 rings of inner welded circular steel rings are designed, and the components are named YH-R0, YH-R1, YH-R2, YH-R3, YH-R4, YH-R5 and YH-R8, respectively. The specific parameters are shown in Table 9

**Table 9:** Quantity-optimized design components.

Sample numbering	Steel pipe size D × t × l (mm)	Number of annular bars	Number of spiral bar rotations	Stirrup spacing (mm)
YH-R0	370 × 10 × 1000	0	0	/
YH-R1	370 × 10 × 1000	1	0	500
YH-R2	370 × 10 × 1000	2	0	333
YH-R3	370 × 10 × 1000	3	0	250
YH-R4	370 × 10 × 1000	4	0	200
YH-R5	370 × 10 × 1000	5	0	166
YH-R8	370 × 10 × 1000	8	0	111

When the influence analysis of steel bar spacing is considered, the number of welded steel rings is three-ring steel bars, and the spacing is designed to be 250 mm, 300 mm, 350 mm, 400 mm and 450 mm. A circular steel bar in the middle of the three rings is in the middle of the steel pipe height. Each component is named R3-250, R3-300, R3-350, R3-400 or R3-450, and the specific parameters of the component are shown in Table 10.

**Table 10:** Pitch optimization design components.

Sample numbering	Steel pipe size D × t × l (mm)	Number of annular bars	Number of rotations of the spiral bars	Stirrup spacing (mm)
R3-250	370 × 10 × 1000	3	0	250
R3-300	370 × 10 × 1000	3	0	300
R3-350	370 × 10 × 1000	3	0	350
R3-400	370 × 10 × 1000	3	0	400
R3-450	370 × 10 × 1000	3	0	450

**Fig. 22:**
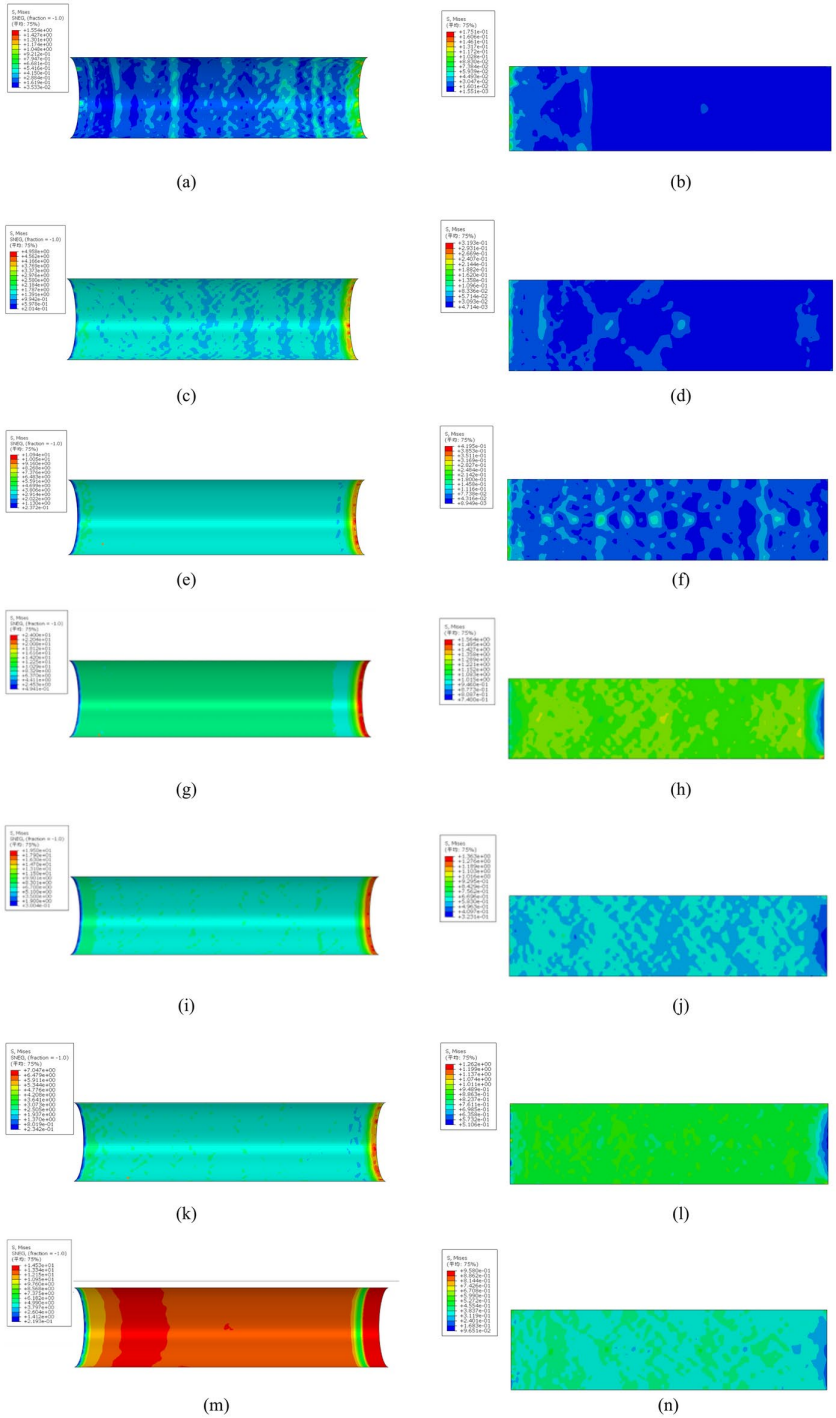
Quantity-optimized equivalent force maps for each component. (**a**) Stress contour of the inner wall profile of the steel pipe of YH-R0, (**b**) Concrete stress contour of YH-R0, (**c**) Stress contour of the inner wall profile of the steel pipe of YH-R1, (**d**) Concrete stress contour of YH-R1, (**e**) Stress contour of the inner wall profile of the steel pipe of YH-R2, (**f**) Concrete stress contour of YH-R2, (**g**) Stress contour of the inner wall profile of the steel pipe of YH-R3, (**h**) Concrete stress contour of YH-R3, (**i**) Stress contour of the inner wall profile of the steel pipe of YH-R4, (**j**) Concrete stress contour of YH-R4, (**k**) Stress contour of the inner wall profile of the steel pipe of YH-R5, (**l**) Concrete stress contour of YH-R5, (**m**) Stress contour of the inner wall profile of the steel pipe of YH-R8, (**n**) Concrete stress contour of YH-R6.

Based on this component construction method, seven components are established to analyze the influence of the number of internally welded circular steel bars. Figure 22 shows a diagram of the equivalent stress distribution between the inner side of the steel pipe and the outer surface of the core concrete, where the loading displacement of each component is 15 mm. Moreover, the peak and trough values of the inner layer stress of the steel pipe and the extreme stress values of the core concrete surface layer in the model are summarized in Table 11.

**Table 11:** Maximum value of the component stress (MPa).

Sample number	Maximum value of the inner wall of the steel pipe	Minimum value of the inner wall of the steel pipe	Maximum value of the concrete surface	Minimum surface of the concrete surface
YH-R0	1.554	0.03533	0.1751	0.001551
YH-R1	4.958	0.2014	0.3193	0.004714
YH-R2	10.94	0.2372	0.4195	0.008949
YH-R3	24.00	0.4941	1.564	0.7400
YH-R4	19.50	0.3004	1.363	0.3231
YH-R5	7.047	0.2342	1.262	0.5106
YH-R8	14.53	0.2193	0.958	0.0965

The analysis in the above chart reveals that the structural measures of the internally welded steel bar greatly increase the bonding stress at the contact interface between the steel pipe and the concrete when it is launched, and the maximum stress values of the inner wall of the steel pipe and the core concrete surface of the steel pipe increase from 1.554 MPa and 0.1751 MPa to 4.958 MPa and 0.3193 MPa, respectively, compared with those of component YH-R0 after the circular steel bars are welded. However, the bonding stress of the contact interface does not increase with increasing number of rings of circular steel bars welded inside, but a fixed number of welds cause the interface bonding stress to reach the maximum value, and the bonding strength decreases after this number is exceeded^33^. The bonding strength of the component begins to decrease after the number of welding turns exceeds 3, the bonding stress of the inner wall of the steel pipe is significantly lower than that of the component welded with 4 circular steel bars, and the stress of the inner wall of the steel pipe of the YH-R8 component when the number of welded steel rings reaches 8 turns is lower than that of the component YH-R3 welded with 3 turns^34–36^.

Figure 22 (m) and (n) show that the structural measures of welding 8-ring reinforcements also increase the average stress of the inner wall of the steel pipe and the surface of the core concrete, which may be due to the gradual extrusion of the constrained core concrete caused by the inner welded reinforcement ring, and the increase in the number of welded reinforcement rings leads to more obvious extrusion; this also shows that the inner welded reinforcement ring can improve the stress distribution of the contact interface during push-out, but it is not that the greater the number of welded rings is, the greater the effect^37^.

Overall, the stress values of YH-R3 and YH-R4 are better than those of YH-R5 and YH-R8 when they are introduced, and the stress values of the contact interfaces of the YH-R3 members are the greatest.

**Fig. 23:**
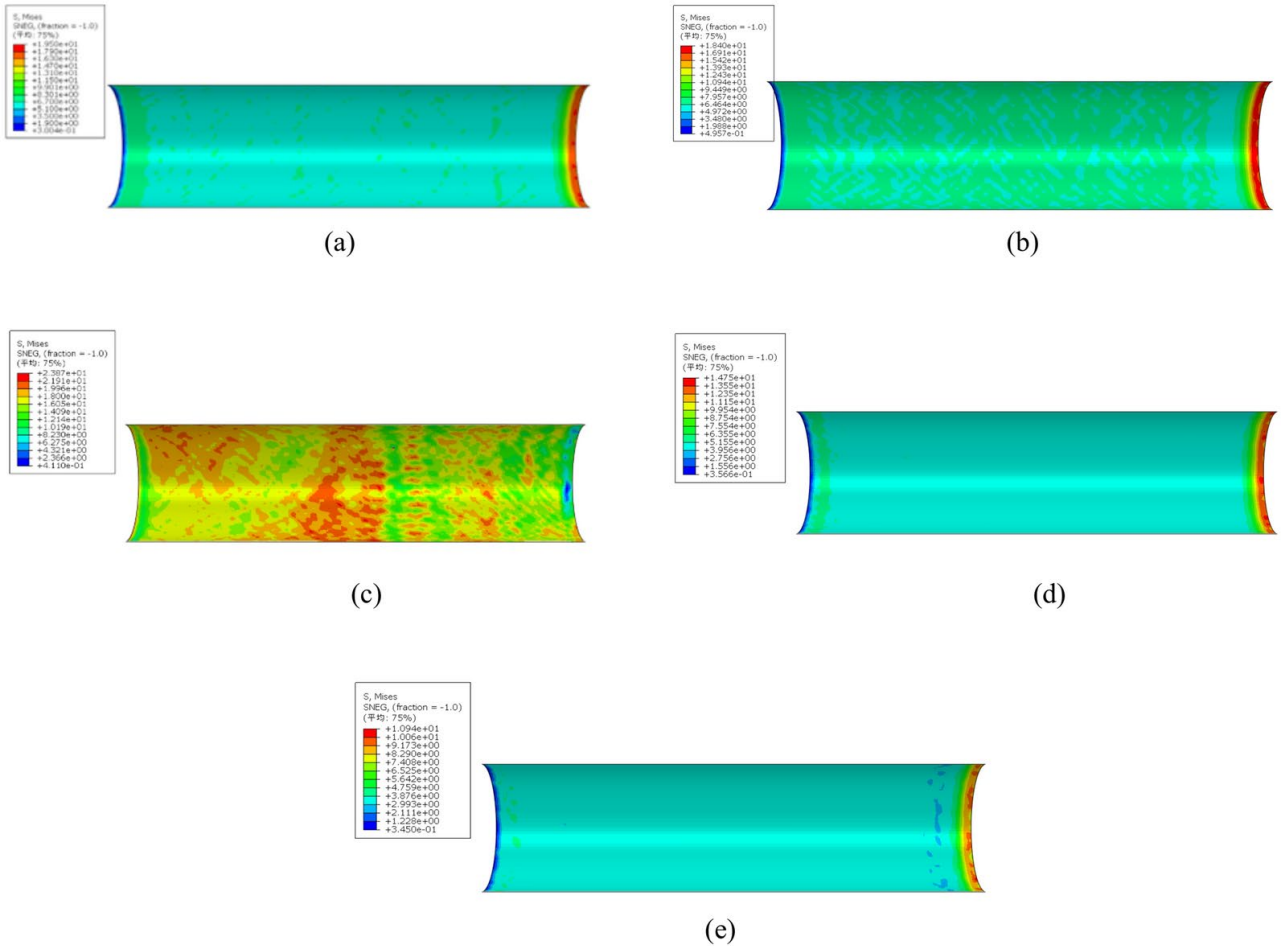
Quantity-optimized equivalent force maps for each component. (**a**) Stress contour of the inner wall profile of an R3-250 steel pipe, (**b**) Stress contour of the inner wall profile of an R3-300 steel pipe, (**c**) Stress contour of the inner wall profile of an R3-350 steel pipe, (**d**) Stress contour of the inner wall profile of an R3-400 steel pipe, (**e**) Stress contour of the inner wall profile of an R3-450 steel pipe.

To analyze the influence of the spacing of the inner welded circular steel bars, five kinds of components are established, and the stress of the contact interface when the loading displacement of each component is 15 mm is also analyzed. The equivalent stress contour diagram of the inner wall of the steel pipe of each component is shown in Fig. 23, and the maximum and minimum stress values of the inner surface of the steel pipe of the model at this time are listed in Table 12^38^.

**Table 12:** Maximum value of the component stress (MPa).

Sample number	Maximum value of the inner wall of the steel pipe	Minimum value of the inner wall of the steel pipe
R3-250	19.50	0.3004
R3-300	18.40	0.4957
R3-350	23.87	0.4110
R3-400	14.75	0.3566
R3-450	10.94	0.3450

The analysis shows that the change in the spacing of the inner welded circular steel bar has little effect on the stress value of the core concrete surface when it is launched but only affects the position of the concrete when it is extruded. Figure 23 and Table 12 show that the change in the spacing of the inner welded steel bars continuously affects the stress distribution state of the inner wall of the steel pipe. The stress of the inner wall of the steel pipe reaches a maximum of 23.87 MPa for the R3-350 member with a spacing of the internally welded steel bars of 350 mm, and the stress distribution state of the inner wall of the steel pipe also reaches the best value. Therefore, R3-350 is the optimal spacing of the welded steel bars with a spacing of 350 mm.

## Conclusion

Optimization measures for internally welded steel bars are proposed to improve the bond strength and vibration resistance of concrete-filled steel tubes, and the main conclusions are as follows.

1. Self-compacting concrete increases the amount of expansion agent, and pumping and vacuum assist in pouring. The concrete in the pumping jacking process achieves uniform filling via the continuous flow of jacking force and removes the effect of internal bubbles, but the jacking force cause approximately 1/3 of the bubbles to hollow out.
2. Push-out tests were performed on four CFSTs with different internally welded reinforcements. The following test results were analyzed: failure modes, load‒slip, bond failure load, load‒strain, strain distribution, and slip state. These measures increase the bond strength and reduce the vibration impact. The maximum bearing capacity of R1 is > 3 times that of R0, and energy consumption doubles. More reinforcement rings indicate greater bond strength and energy dissipation. R3’s adhesion > R2’s > R1’s. These measures lessen the vibration effect on components according to the final push-out length analysis.
3. The results demonstrate that the best effect is achieved when the number of circular steel bars is welded for three turns and the spacing is 350 mm. The results also show that the optimization measures of the internally welded steel bar can optimize the stress of the concrete-filled steel tube arch rib, providing a reference for practical engineering applications.
4. Internal welded reinforcement plays a key role in reducing void defects by improving the bonding between steel and concrete, which helps transfer stresses and prevents voids or cracks due to stress concentration. This enhances the overall stability and integrity of the structure, limits crack propagation, and improves crack resistance. Welded reinforcement also reduces moisture and corrosive infiltration, lowering the risk of corrosion and enhancing durability. It helps control temperature differences, mitigating environmental impacts. Additionally, it ensures precise placement during construction, reducing defects. In summary, welded reinforcement significantly reduces void defects and improves structural durability.

## Data Availability

All data generated or analyzed during this study are included in this published article [and its supplementary information files].
